# Factors affecting failed induction of labour among Indian women

**DOI:** 10.6026/9732063002001424

**Published:** 2024-10-31

**Authors:** Vaishnavi S Raghoji, Rajkumar P Patange, Supriya Patil

**Affiliations:** 1 Department of Obstetrics and Gynecology, Krishna Institute of Medical Sciences, Karad - 415110, Maharashtra, India

**Keywords:** Forced internal labor (FIOL), labor induction, filed induction, successful induction, oligohydramnios (OGD), questionnaire, clinical assessment, laboratory findings

## Abstract

Medical personnel are typically worried when an induction of labour fails. Therefore, it is of interest to evaluate the factors leading to forced internal labor (FIOL). Hence, 92 patients were divided into 2 groups namely, group A - filed induction and group B - successful induction to collect information with the help of questionnaire, clinical assessment and laboratory findings. We found that, the presence of oligohydramnios (OGD) does not significantly impact the success of labor induction between the 2 groups (p = 0.459). Thus, we show the importance of considering age, parity, gestational age, bishop score and body mass index for optimizing labor induction.

## Background:

Induction of labor (IOL) is a commonly used procedure in modern obstetrics. It involves the artificial stimulation of uterine contractions after 28 weeks of gestation, but before labor begins naturally, with the goal of facilitating a vaginal delivery [1, 2]. It is only when the benefits of terminating the pregnancy outweigh the risks of continuing it that an IOL, a potentially life-saving obstetric procedure, is advised [3]. The selection of induction techniques, whether they involve medication, mechanical methods, or a combination of both, can play a crucial role in determining the outcome of the induction process. There are several commonly used techniques for labor induction, including the mechanical approach using an ARM and balloon catheter, as well as the pharmacologic approach using oxytocin and misoprostol [1, 4]. However, a variety of suggested criteria for detecting FIOL exist, including the mode of delivery (cesarean versus vaginal) and precise time intervals in which the active phase of labor is achieved or an adequate number of uterine contractions [5]. The Federal Ministry of Health of Ethiopia (FMOH) defines failure to generate adequate uterine contractions (3-5C/10min/≥40s) after 6 to 8 hours of oxytocin infusion at the maximal dosage as FIOL [6]. On the other hand, the majority of other studies defined FIOL as the inability to give birth vaginally or by cesarean section (CS) [7, 8].

Approximately 20% of pregnancies globally are affected by IOL2, with around 20% of these pregnancies ultimately leading to cesarean section deliveries [9]. In industrialized nations, around 25% of deliveries involve induction of labor (IOL). Interestingly, in some developing countries, the rate of IOL is comparable to that of developed nations, ranging from 1.4% in Nigeria to 35.5% in Sri Lanka. According to a source cited as [10]. It is widely acknowledged that IOL plays a crucial role in reducing maternal morbidity and mortality associated with pregnancy and its complications. Unfortunately, IOL doesn't always yield the desired results. In some cases, it can lead to emergency cesarean delivery (CS delivery), which has been associated with various negative consequences for both the mother and the newborn. These include postpartum hemorrhage (PPH), hysterectomy; wound complications, sepsis [11], injuries to the newborn, maternal death [12, 13] and longer recovery periods [14, 15]. Induced labor is associated with a greater likelihood of caesarean sections and other surgical deliveries when compared to spontaneous labor in women [12]. According to a study done at Mattu Karl Hospital in Ethiopia, FIOL had a negative impact on 6.5% of women and 35.5% of neonates [12]. Therefore, it is of interest to report the prevalent factors associated with failed induction of labour (FIOL).

## Materials and Methods:

The current retrospective observational study was conducted in the department of obstetrics and gynecology at Krisna Vishwa Vidyapeeth Hospital starting from June 2022 ending to November 2023 with a total of 92 patients. These patients were further divided into 2 groups, *i.e.,* group A included cases with filed induction, while group B included cases with successful induction with 46 patients each, respectively. Upon admission for IOL, eligible women were approached for participation. Data was collected with the help of structured questionnaire which includes demographic data, obstetric history and details of current pregnancy, clinical assessment includes pre-induction bishop score, method of induction used Dinoprostone gel (PGE2), the gel can be used 2 times, maternal vital sign, general condition, cervical status, membrane status and review of medical records with the help of lab investigation which include routine antenatal investigation, specific investigation as indicated (*eg:* glucose tolerance test for GDM, BP monitoring for high BP disorder), fetal outcome(FO) includes APGAR score, birth weight, neonatal complication and NICU admission if required and maternal outcome(MO) includes mode of delivery (MOD), complication during labour and postpartum recovery(PPR).

## Inclusion criteria:

[1] Who all were admitted for IOL

[2] Singleton PG.

[3] Gestational age (GA) ≥ 28 weeks.

[4] Cephalic presentation.

[5] Consent provided for participation.

## Exclusion criteria:

[1] Multiple GA.

[2] Known fetal anomalies.

[3] Previous uterine surgery (*e.g.* cesarean section, myomectomy).

[4] Severe medical complications requiring immediate delivery.

[5] Non-cephalic presentation.

[6] Placental abnormalities (*e.g.* placenta previa).

## Statistical analysis:

Data was analyzed using SPSS software. Comparative analysis between group A & B was done using chi-square test for categorical variables and t-test for continuous variables. Multivariate logistic regression was identified using in depend predictors of filed induction.

## Results:

Table 1 shows that, in each group 46 cases were enrolled. Among those aged <20 years, 13 cases (28.3%) resulted in filed induction compared to 4 cases (8.7%) that were successful induction. In the 20-30 years age group, 30 cases (65.2%) filed induction while 37 cases (80.4%) were successful induction. For individuals aged 31- 40 years, 3 cases (6.5%) filed induction and 5 cases (10.9%) were successful induction. P value is less than 0.05 it means that filed induction is higher in lower age group (<20 years) compare to higher age groups. (P = 0.049). Table 2 shows that, among nullipara, 28 cases (60.9%) experienced filed induction compared to 18 cases (39.1%) that resulted in successful induction. Conversely, in the multipara group, 18 cases (39.1%) had filed induction while 28 cases (60.9%) were successful induction. The P value is less than 0.05 it indicating a higher rate of successful induction among multipara compared to nullipara women. (P = 0.037). Table 3 shows that, among pregnancy at less than 37 weeks gestational age, 18 cases (39.1%) resulted in filed induction compared to 4 cases (8.7%) that were successful induction. In the 37-40 weeks gestational age group, 25 cases (54.3%) filed induction while 38 cases (82.6%) were successful induction. For pregnancy beyond 40 weeks, 3 cases (6.5%) experienced filed induction and 4 cases (8.7%) were successful induction. The P value is less than 0.05 it means that higher rates of successful induction among age at 37 to 40 weeks compared to other groups. (P = 0.002). Table 4 shows that, among cases with a FV-BS, 5 (10.9%) experienced filed induction while 39 (84.8%) were successful induction. In contrast, among cases with a U-FV-BS, 41 (89.1%) resulted in filed induction and 7 (15.2%) were successful induction. The P value is less than 0.05 it indicating higher success rates in cases with a FV-BS and a significant association between U-FV scores and filed induction (P = 0.030).

Table 5 shows that, in N-BMI group, 6 cases (13.0%) experienced filed induction compared to 31 cases (67.4%) that were successful induction. In the OW group, 26 cases (56.5%) filed induction while 11 cases (23.9%) were successful induction. Among the OB group, 14 cases (30.4%) had filed induction and 4 cases (8.7%) were successful induction. The P value is less than 0.05 it means that the result is statistically significant. In OW and OB group, the rate of filed induction is higher compare to N-BMI group. (P = <0.001). Table 6 shows that, among cases with intact membranes, 40 (87.0%) experienced filed induction while 39 (84.8%) were successful induction. Conversely, among cases with ruptured membranes, 6 (13.0%) had filed induction and 7 (15.2%) were successful induction. The P value is more than 0.05 it means the results is statistically not significant (P=0.764). Table 7 shoes that, among cases where HYT-D of pregnancy was present, 18 (39.1%) experienced filed induction while 22 (47.8%) were successful induction. Conversely, among cases where HYT-D of pregnancy was absent, 28 (60.9%) resulted in filed induction and 24 (52.2%) were successful induction. The P value is more than 0.05 it means the result is statistically not significant (P=0.401). Table 8 shows that, among cases where GDM was present, 4 (8.7%) experienced filed induction while 2 (4.3%) were successful induction. In contrast, among cases where GDM was absent, 42 (91.3%) resulted in filed induction and 44 (95.7%) were successful induction. This result suggests that the presence of GDM does not significantly impact the success of labor induction (P=0.398).

Table 9 shows that, in cases with IUFG, 4 (8.7%) experienced filed induction while 2 (4.3%) were successful induction. Conversely, among cases with normal IUGR, 42 (91.3%) resulted in filed induction and 44 (95.7%) were successful induction. Thus, suggests that IUFG status does not significantly impact the success of labor induction, as indicated by comparable rates of successful induction in both groups. (P =0.398). Table 10 shows that among cases where induction failed, 1 (2.2%) resulted in IU-D compared to 3 (6.5%) cases that were successful induction. In contrast, among cases where induction was successful, 45 (97.8%) resulted in FS while 43 (93.5%) were successful induction. The result is statistically not significant. (P= 0.308). Table 11 shows that, among cases where OGD was present, 5 (10.9%) experienced filed induction while 3 (6.5%) were successful induction. However, in cases where OGD was absent, 41 (89.1%) resulted in filed induction and 43 (93.5%) were successful induction. The P value is >0.05 it indicates that the presence of OGD does not significantly impact the success of labor induction. (P = 0.459).

## Discussion:

46 cases were enrolled in each group in the present study. Among those aged less than 20 years, 13 cases (28.3%) resulted in filed induction compared to 4 cases (8.7%) that were successful induction. In the 20-30 years age group, 30 cases (65.2%) filed induction while 37 cases (80.4%) were successful induction. For individuals aged 31 - 40 years, 3 cases (6.5%) filed induction and 5 cases (10.9%) were successful induction. P value is less than 0.05 it means that filed induction is higher in lower age group (<20 years) compare to higher age groups. (P = 0.049) Similar result observed in the study by Tadesse *et al.* [11] and Demssie *et al.* [16]. In the research of Tadesse *et al.* among women aged 30 and younger, 105 (19.3%) had filed induction, whereas 439 (80.7%) had successful ones. Among women over the age of 30, 69 (40.8%) had unsuccessful induction (US-I), whereas 100 (59.2%) had a successful one. demonstrating a much-increased chance of US-I in this age range. After accounting for possible confounders, the adjusted odds ratio (AOR) remains considerably higher at 3.7 (95% CI: 2.2-6.2), suggesting that advanced maternal age (> 30 years) is independently linked with a greater risk of US-I of labour. P < 0.001 there is a significantly substantial connection between age above 30 years and failure induction of labour [17]. EA *et al.* found individuals aged <20 years exhibited a notably higher incidence of filed induction (39.6%) compared to older counterparts (20-34 years, 24.8%; 35-49 years, 20.9%). A significant association was observed with those under 20 years demonstrating increased odds of filed induction (COR 2.47, 95% CI 1.09-5.57, p < 0.05) relative to the reference group (20-34 years). Conversely, the result was not statistically significant, it means no differences were found in induction outcomes between the 20-34 years and 35-49 years age groups [16].

Among nullipara, 28 cases (60.9%) experienced filed induction compared to 18 cases (39.1%) that resulted in successful induction. Conversely, in the multipara group, 18 cases (39.1%) had filed induction while 28 cases (60.9%) were successful induction. The P value is less than 0.05 it indicating a higher rate of successful induction among multipara compared to nullipara women. (P = 0.037) Tadesse *et al.* study found that nullipara women had a higher risk of FIOL than multipara women [11]. In present study, in Normal BMI group, 6 cases (13.0%) experienced filed induction compared to 31 cases (67.4%) that were successful induction. In the OW group, 26 cases (56.5%) filed induction while 11 cases (23.9%) were successful induction. Among the Obese group, 14 cases (30.4%) had filed induction and 4 cases (8.7%) were successful induction. The P value is less than 0.05 it means that the result is statistically significant. In OW and OB group, the rate of filed induction is higher compare to Normal BMI group. (P = <0.001) Additionally, among cases with intact membranes, 40 (87.0%) experienced filed induction while 39 (84.8%) were successful induction. Conversely, among cases with ruptured membranes, 6 (13.0%) had filed induction and 7 (15.2%) were successful induction. The P value is more than 0.05 it means the results is statistically not significant. (P=0.764) Moreover, among cases where filed induction, 1 (2.2%) resulted in IU-D compared to 3 (6.5%) cases that were successful induction. In contrast, among cases where induction was successful, 45 (97.8%) resulted in fetal survival while 43 (93.5%) were successful induction. The result is statistically not significant. (P= 0.308) Ejigu *et al.* found women with BMI >24 kg/m^2^ (49 cases) had significantly higher odds of failed induction compared to those with BMI ≤24 kg/m^2^ (43 cases) (AOR 5.71, 95% CI 3.26-10.01, p < .001) [17]. Ehrenberg *et al.* discovered that intrauterine foetal development status had no significant impact on labour induction outcomes [18]. Similarly, Tanir *et al.* found that, whereas IUGR is related with various problems, it has no significant effect on labour induction success rate [19]. Similarly, Grobman *et al.* discovered that, while inducement of labour can raise risks, the overall impact on FS did not differ substantially between unsuccessful and successful induction however [20]. Zhang *et al.* pointed out those unsuccessful inductions might result in greater rates of caesarean birth, which could have an indirect influence on infant outcomes [21].

In the multivariable analysis of factors influencing filed induction of labor at AHMC, Ethiopia in 2020, the presence of Oligohydramnios did not show a significant association with the likelihood of filed induction. The data revealed that among pregnancy without OGD, 25.4% experienced filed induction compared to 26.2% in cases with OGD (AOR 1.0, 95% CI 0.7-1.7). This finding suggests that OGD may not independently impact the success of labor induction in this cohort. However, further investigation with larger sample sizes or specific clinical contexts may be needed to better understand its potential influence on induction outcomes [22]. His duration of induction is also a known risk factor. The risk increases linearly during an induction, with more vaginal births happening early on and more caesarean deliveries occurring later [23]. In Beckmann's 2007 study, the length of the latent period dramatically increased the risk of a C-section delivery [24]. Certain fetal features may also influence induction success. Higher birth weights have been linked to an increased risk of US-I, including a higher caesarean delivery rate and a lower vaginal delivery rate [24, 25].

## Conclusion:

Body mass index (BMI) was a key determinant. Normal BMI is associated with higher success rates compared to overweigh and obese categories. Cervical ripening prior to induction was significantly beneficial, improving success rates. Membrane status, hypertensive disorder, gestational diabetes mellitus, fetal growth restriction, intrauterine death and oligohydramnio showed no significant impact on induction success.

## Figures and Tables

**Table 1:** Age distribution

	**Failed Induction(FI)**		**Successful Induction(SI)**		
**Age (years)**	**Cases**	**%**	**Cases**	**%**	**P value**
< 20	13	28.30%	4	8.70%	
20-30	30	65.20%	37	80.40%	0.049
31-40	3	6.50%	5	10.90%	
Total	46	100.00%	46	100.00%	

**Table 2:** Parity distribution

**Parity**	**FI**		**SI**		
	**Cases**	**%**	**Cases**	**%**	**P value**
Nullipara (NP)	28	60.90%	18	39.10%	
Multipara(MP)	18	39.10%	28	60.90%	0.037
Total	46	100.00%	46	100.00%	

**Table 3:** GA distribution

**Parity**	**FI**		**SI**		
	**Cases**	**%**	**Cases**	**%**	**P value**
Nullipara(NP)	28	60.90%	18	39.10%	
Multipara(MP)	18	39.10%	28	60.90%	0.037
Total	46	100.00%	46	100.00%	

**Table 4:** PIBS distribution

**Pre-induction bishop score (PIBS)**	**FI**		**SI**		
	**Cases**	**%**	**Cases**	**%**	**P value**
Favorable (FV)	5	10.90%	39	84.80%	
Unfavorable(U-FV)	41	89.10%	7	15.20%	0.03
Total	46	100.00%	46	100.00%	

**Table 5:** BMI distribution

**BMI**	**FI**		**SI**		
	**Cases**	**%**	**Cases**	**%**	**P value**
Normal(N)	6	13.00%	31	67.40%	
Overweight(OW)	26	56.50%	11	23.90%	
Obese (OB)	14	30.40%	4	8.70%	< 0.001
Total	46	100.00%	46	100.00%	

**Table 6:** PIMS distribution

**Pre-induction membrane status (PIMS)**	**FI**		**SI**		
	**Cases**	**%**	**Cases**	**%**	**P value**
Intact	40	87.00%	39	84.80%	
Ruptured	6	13.00%	7	15.20%	0.764
Total	46	100.00%	46	100.00%	

**Table 7:** HYT-D comparison

**Hypertensive disorder of pregnancy **	**FI**		**SI**		
**(HYT-D-PG)**	**Cases**	**%**	**Cases**	**%**	**P value**
Present	18	39.10%	22	47.80%	
Absent	28	60.90%	24	52.20%	0.401
Total	46	100.00%	46	100.00%	

**Table 8:** GDM distribution

**GDM**	**FI**		**SI**		
	**Cases**	**%**	**Cases**	**%**	**P value**
Present	4	8.70%	2	4.30%	
Absent	42	91.30%	44	95.70%	0.398
Total	46	100.00%	46	100.00%	

**Table 9:** IUFG distribution

**Intrauterine fetal growth**	**FI**		**SI**		
**(IUFG)**	**Cases**	**%**	**Cases**	**%**	**P value**
IUGR	4	8.70%	2	4.30%	
Normal	42	91.30%	44	95.70%	0.398
Total	46	100.00%	46	100.00%	

**Table 10:** FS distribution

**Fetal Survival (FS)**	**FI**		**SI**		
	**Cases**	**%**	**Cases**	**%**	**P value**
Intrauterine Death (IU-D)	1	2.20%	3	6.50%	
Survived	45	97.80%	43	93.50%	0.308
Total	46	100.00%	46	100.00%	

**Table 11:** OGD distribution

**Oligohydramnios**	**Failed Induction**		**Successful Induction**		
**(OGD)**	**Cases**	**%**	**Cases**	**%**	**P value**
Present	5	10.90%	3	6.50%	
Absent	41	89.10%	43	93.50%	0.459
Total	46	100.00%	46	100.00%	
